# Annual-scale assessment of mid-20th century anthropogenic impacts on the algal ecology of Crawford Lake, Ontario, Canada

**DOI:** 10.7717/peerj.14847

**Published:** 2023-03-08

**Authors:** Matthew G. Marshall, Paul B. Hamilton, Krysten M. Lafond, Nawaf A. Nasser, Francine M.G. McCarthy, R. Timothy Patterson

**Affiliations:** 1 Ottawa-Carleton Geosciences Center and Department of Earth Sciences, Carleton University, Ottawa, Ontario, Canada; 2 Research and Collections, Canadian Museum of Nature, Ottawa, Ontario, Canada; 3 Department of Biology, Queen’s University, Kingston, Ontario, Canada; 4 Department of Earth Sciences, Brock University, St. Catharines, Ontario, Canada

**Keywords:** Acid Deposition, Algae, Anthropocene, Diatoms, Scaled Chrysophytes, Varves, Climate Change, Dissolved Organic Carbon (DOC)

## Abstract

Meromictic Crawford Lake, located in SW Ontario, Canada is characterized by varved sediments, making it suitable for high-resolution paleoecological studies. Freeze cores, the only coring method available that reliably preserves the fragile laminations representative of seasonal deposition in the lake, were used to document siliceous diatom and chrysophyte community structure at an annual resolution from 1930–1990CE. Stratigraphically constrained cluster analysis identified major assemblage changes that are believed to have been caused by local, regional and possibly global anthropogenic impacts. The assemblage changes within the siliceous algae are attributed to regional weather and increased industrial emissions and related effects of acid deposition on the lake’s catchment associated with the Great Acceleration –the massive economic, industrial, and demographic expansion beginning in the mid-20th century. Observed increases in spheroidal carbonaceous particles (SCPs) in varved lake sediment dating to the early 1950s record rapidly expanding steel production without emission controls around 30 km upwind of the lake. The findings reported here reflect major changes in earth systems that the Anthropocene Working Group recommends for a proposed epoch to be termed the Anthropocene, providing support for the laminated sediments from Crawford Lake as a potential Global boundary Stratotype Section and Point (GSSP).

## Introduction

The current geologic epoch, the Holocene, began approximately 11,650 calendar years before present (cal BP), and is defined by the development of relatively warm, stable interglacial conditions (Walker et al., 2009). However, some researchers have argued that the planet has more recently become dominated by unique anthropogenic impacts at global, regional, and local scales, resulting in conditions well beyond the natural variability through the Holocene Epoch (Paerl, 2006; Doney et al., 2009; Zalasiewicz et al., 2010). These impacts have been extensive to the point that this evidence is being permanently incorporated into the geologic record worldwide (Zalasiewicz et al., 2010; Zalasiewicz et al., 2014). Despite considerable semantic baggage associated with the term that has been used loosely since it was coined by Paul Crutzen & Gene Stoermer (Crutzen & Stoermer, 2000; Crutzen, 2002), the Anthropocene Working Group (AWG) of the Sub-commission on Quaternary Stratigraphy has been working toward a formal definition of a new geologic epoch to succeed the Holocene. There has been considerable debate concerning a start date for the proposed Anthropocene, but a consensus was reached to have this new epoch start in the mid-twentieth century (Subramanian, 2019). To do this, an associated series must be identified in the geologic record.

A good candidate for the Anthropocene Global boundary Stratotype Section and Point (GSSP) must preserve changes demonstrative of the global human impacts associated with the Great Acceleration as well as be able to provide a precise and accurate datum for the base of the unit. The GSSP must be accessible and be associated with a site that will preserve these changes through time (Waters et al., 2018; Subramanian, 2019; Head, 2019; Anthropocene Working Group, 2020). Crawford Lake, located within the Crawford Lake Conservation Area, is a good candidate to be the Anthropocene GSSP (McCarthy et al., 2023). This sheltered, relatively deep (∼24 m deep) lake is characterized by permanent stratification between the upper and lower parts of the water column at ∼15.5m depth. The deeper, more electrically conductive, and unusually well-oxygenated monimolimnion inhibits disturbance of the sediment-water interface by forces such as wave action, bioturbation and mobilization leaching (Llew-Williams, 2022). The varved sediments spanning the Canadian zone (since 1867 CE), as well as the interval of Indigenous Agricultural settlement between the 13th and latest 15th/earliest 16th centuries, allow for paleolimnological studies at resolutions unmatched by studies of more homogenized core material that depend on radioisotope core dating methodologies (Clark & Royall, 1995; McAndrews & Boyko-Diakonow, 1989; Ekdahl et al., 2007; McAndrews & Turton, 2008). For dating sediments using radioisotope methodologies, where uncertainties within the calculated age-depth models are based on the number of half-lives and isotope activity within the sample, there are often errors of precision. Production of artificial radiocarbon by nuclear explosions into the upper atmosphere present difficulties when trying to define a GSSP based in the mid-20th century to the year. However, in tandem, the annual resolution of varved sediments coupled with isotope dating makes Crawford an excellent GSSP candidate.

**Figure 1:**
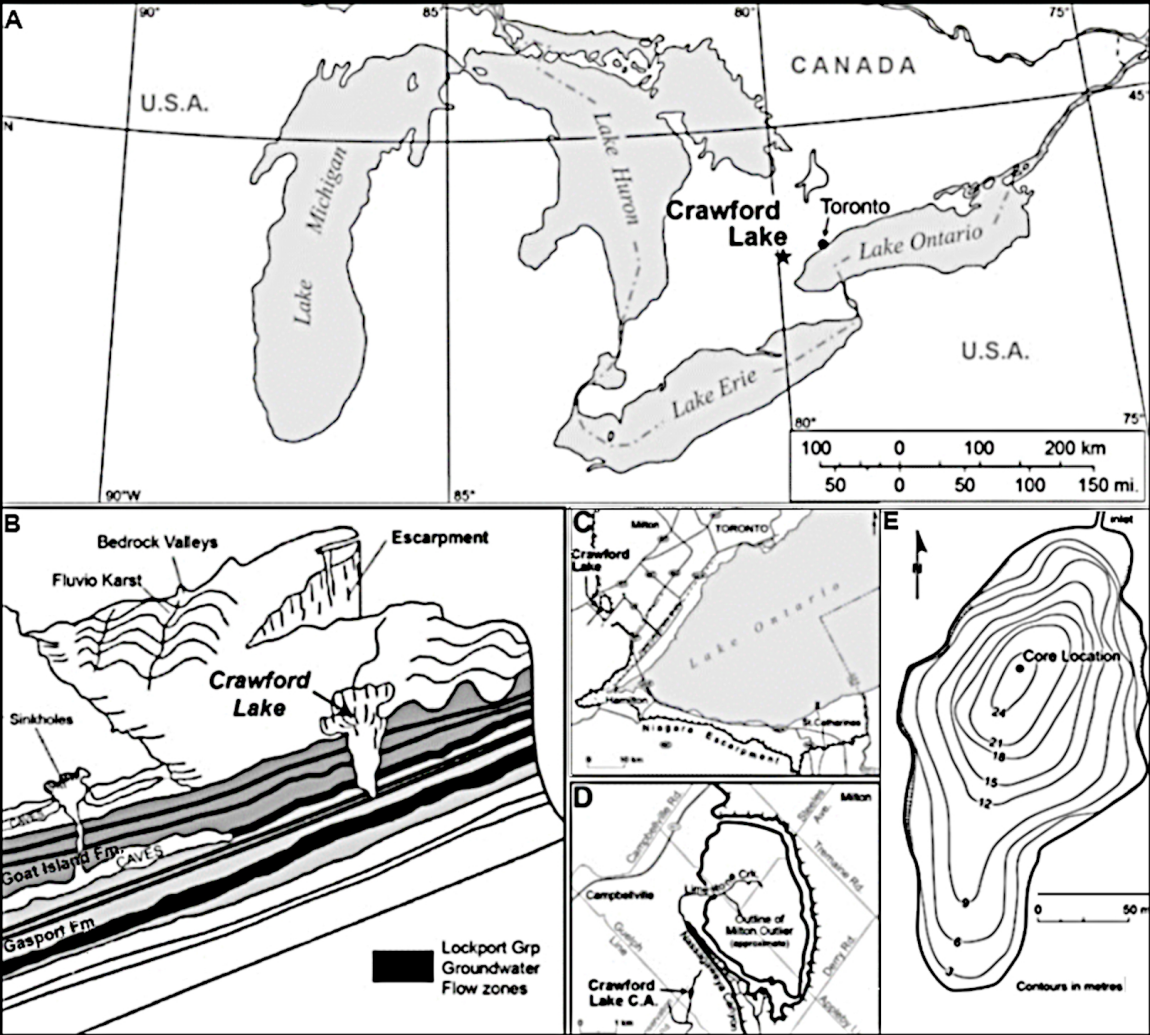
Site location. (A) Map depicting Crawford Lake in relation to Toronto within Ontario, Canada. (B) Situation of Crawford Lake relative to local geological formations. (C, D) Location of Crawford Lake locally. (E) Core location and general bathymetry within Crawford Lake. Bathymetry is adapted with permission from Boyko (1973).

**Figure 2:**
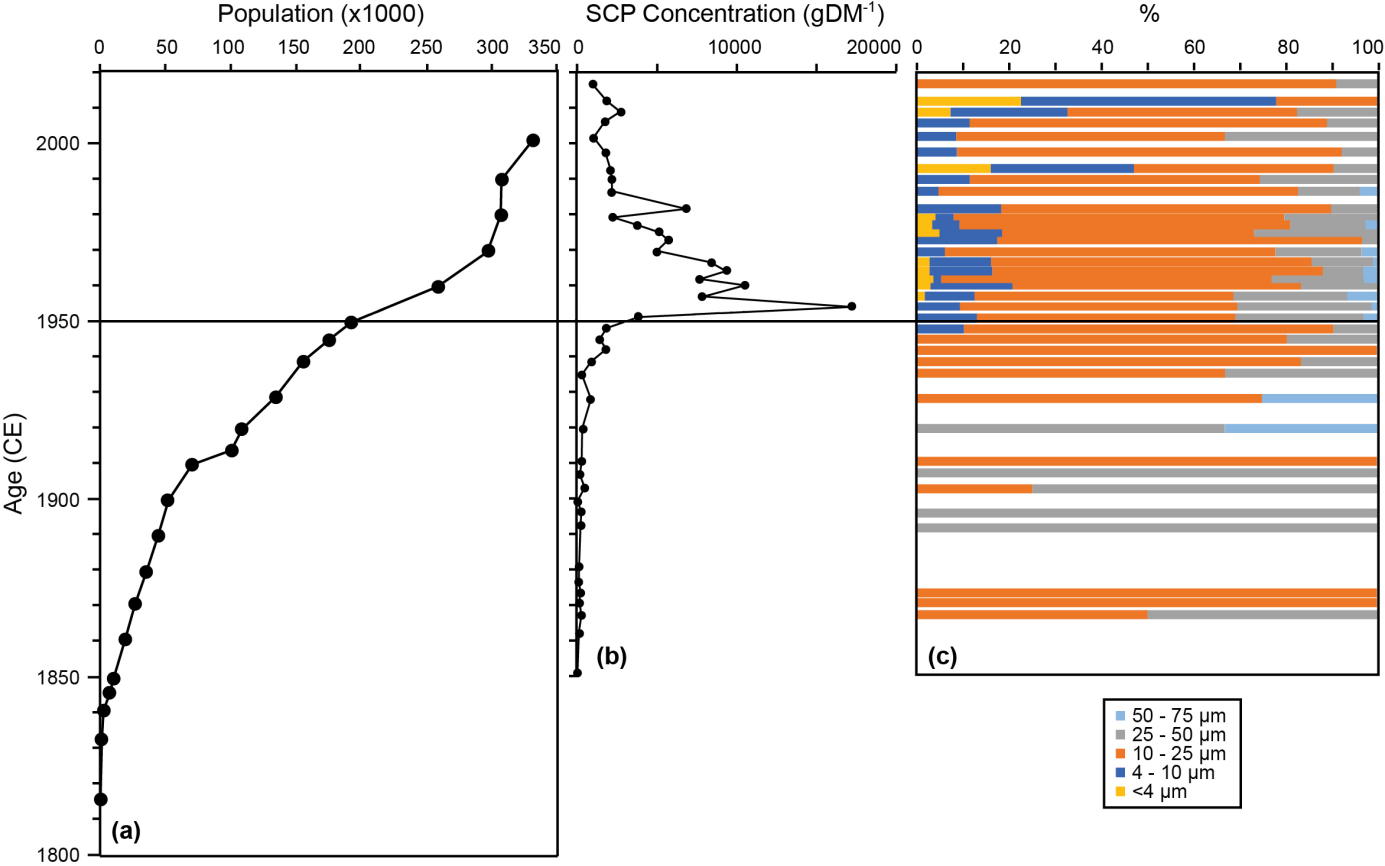
Spheroidal carbonaceous particle (SCP) concentration data. Relationship between the population of Hamilton, Ontario (‘Steeltown’, 303 m upwind from Crawford Lake) and the concentration of spheroidal carbonaceous particles (SCPs) in freeze core CRA19-2FT-B2 from ∼22 m water depth. (A) Population of the nearby town of Hamilton (number of people × 1,000). (B) Spheroidal carbonaceous particle (SCP) concentration in number of particles per gram of dry mass. (C) Composition of SCPs by size fraction percentage. Modified from McCarthy et al. (2023).

Further strengthening Crawford Lake’s position as a record of anthropogenic impacts is its proximity to industrialized areas of the Great Lakes basin, including Hamilton, a city that was built on the steel industry that boomed during the mid-20th century. These changes were concurrent with similar global increases in industrialization that occurred in the post-war era and which garnered Hamilton with the nickname of “Steeltown” (Fig. 1; Arnold, 2007; Arnold, 2012). Despite the lake being situated in a conservation area within a UNESCO World Biosphere Reserve, a variety of airborne anthropogenic markers accumulated within the lake sediments. These airborne markers include contaminants such as sulfur and nitrogen oxides from industrial applications, as well as spheroidal carbonaceous particles (SCPs) created by the burning of fossil fuels, making them a suitable proxy for the increased population, industrialization, and fuel consumption representative of the Great Acceleration—the large increase of industrialization and human population beginning in the mid-20th century (Steffen et al., 2011). These have been suggested to be ideal proxies to mark the base of the Anthropocene (Ruppel et al., 2013; Rose, 2015; Swindles et al., 2015). The first notable presence of SCPs in sediments from Crawford Lake was in the 1930s with a step wise increase during the 1940s (Fig. 2, McCarthy et al., 2023). A massive spike (20-fold) increase was observed in 1952, immediately followed by a 50% drop to 8,000–10,000 gDM^−1^. After 1960, there was a steady decline through to 1980, with a smaller spike ca. 1982 (Fig. 2). The remaining period from 1982 to 2019 showed a continued decline to concentrations <1,000 gDM^−1^.

Previous research has used the varved sediment record in Crawford Lake to unravel its paleolimnological history with many different bio-indicators. For example, pollen, charcoal, chrysophyte cyst and diatom records have provided important information on the effects of past anthropogenic impacts (Ekdahl et al., 2007; Ekdahl et al., 2004; McAndrews & Boyko-Diakonow, 1989; Rybak & Dickman, 1988). Evidence in the lake sediments of eutrophication, nearby fires, and pollen composition has revealed the impacts of Indigenous habitation adjacent to the lake during the late 1200s to 1400s, as well as later 19th century European settlement (Clark & Royall, 1995; McAndrews & Boyko-Diakonow, 1989; McAndrews & Turton, 2008). Other surveys have investigated changes in tree coverage and climate throughout the area, with such studies focusing on the past ∼1,000 years (Yu, 1997; Yu & Eicher, 1998; Yu & Wright, 2001). Despite the plethora of research carried out at Crawford Lake, very few studies have taken advantage of the annual- resolution record afforded by the varved sediments to examine the siliceous microfossils at annual resolution, with only Ekdahl et al. (2004); Ekdahl et al. (2007) having studied diatoms at this resolution, and no studies to this date having studied chrysophyte scales. Gushulak et al. (2021) did however examine both chrysophyte scales and diatom assemblages spanning the 19th and 20th centuries at sub-decadal resolution, showing major assemblage changes concomitant with the proposed Anthropocene lower boundary.

Based on the success of Gushulak et al. (2021) in demonstrating the record of siliceous algae as a robust proxy for documenting paleoenvironmental change within Crawford Lake, and the annual-resolution varve samples collected using the chronology published in McCarthy et al. (2023), the research presented here was carried out to assess changes in scaled chrysophyte and diatom communities at annual resolution across the proposed Anthropocene-Holocene boundary to determine more precisely the paleolimnological onset and impact of the Great Acceleration in Crawford Lake (1930–1990). More specifically, an annual resolution of 20th century ecological changes in a relatively protected system may have implications for policy makers and planners as they develop strategies to manage water resources and ecosystems in an age of massive human population growth, resource consumption, and industrial expansion.

## Materials & Methods

### Study area

Crawford Lake (43°28′06″N, 79°56′55″W), situated on dolomitic bedrock within the Crawford Lake Conservation Area in Milton, Ontario, features a maximum water depth of ∼24 m, surface area of approximately 2.4ha, and is at an elevation of 286 m above sea level. The deflection of wind from nearby forests comprised of *Thuja*, *Betula*, *Ulmus*, *Quercus*, and *Pinus* (McAndrews & Boyko-Diakonow, 1989) and proximity to the Niagara Escarpment with highly alkaline groundwater inputs (Llew-Williams, 2022), as well as the general shape and depth of this karst basin, provides a sheltered environment conducive to meromixis. This environment, with non-mixing but well-oxygenated bottom waters below a sharp chemocline at ∼15.5 m water depth with distinctive pH and conductance conditions, impedes lake bottom bioturbation (Heyde, 2021), allowing seasonal varves to accumulate undisturbed on the lakebed. Light-colored layers formed of calcite precipited in the epilimnion during the summer and predominantly authigenic organic matter accumulates the rest of the year, although primarily during fall turnover (Dickman, 1979; Dickman, 1985; Llew-Williams, 2022) forming the dark laminae. Previous research shows the lake to be alkaline, with high specific conductance but pH lower than 7 in the monimolimnion (Ekdahl et al., 2007; Gushulak et al., 2021; Llew-Williams, 2022).

The climate in the Halton region of southern Ontario is humid continental, with average yearly air temperature ∼7.3 °C (median 7.6) and mean annual precipitation of ∼650 mm (median 632 mm) between the years 1937 and 1990 (Toronto Airport, Ontario: Environment Canada, 2020). Toronto Airport is 35 km east of Crawford Lake. There were 12 years with elevated total summer rainfall >400 mm and one year (1945) with >500 mm. Average annual snow fall from November to March 1937–1990 was 127 mm (median 125) with two years of heavy snow >200 mm, 1950 and 1972.

### Sample collection

A two-faced freeze core was collected (CRA19-2FT-B2), 83 cm in length, in February of 2019 by RTP and associates, using ethanol and dry ice to freeze sediment along the length of a flat faced metal prism. The collection method was similar to that described in Glew, Smol & Last (2001). The core was collected at a depth of ∼22 m, with the sampling location shown in Fig. 1, and transported frozen to Carleton University for subsequent processing and analysis. A scalpel was used to isolate and subsample adjacent light and dark bands varve couplets deposited each calendar year. Each varve-year was matched to the corresponding Gregorian calendar year using known climate phenomena within the area, such as thick varves representative of the Dust Bowl, and were then counted forward and backward temporally using distict varves which are well-preserved within the slabs of frozen sediment. This varve age model was confirmed by analysis of radionuclides whose distribution in the varved sediment mirrors nuclear fallout (McCarthy et al., 2023). Sixty-one subsamples, corresponding to the years 1930 to 1990 CE were prepared for analysis. Diatom analysis was restricted between 1930 to 1980 CE.

### Siliceous algae analysis

Each subsample was processed for siliceous algae following a protocol similar to Battarbee et al. (2001). For this methodology, ≤0.1 g of sample from each varve-identified year was treated with 10% HCl to remove carbonates, and then washed with distilled water numerous times (∼10 washes) until the supernatant reached the pH of the distilled water, to reduce the concentration of dissolved calcium, and neutralize the pH. These samples were then treated with 30% H_2_O_2_ to remove organic matter, and subsequently put through a series of washes until reaching the pH of the distilled water used for washes. These siliceous slurries were then pipetted, following a series of $ \frac{1}{2} $ dilutions until an appropriate siliceous microfossil density was reached, onto coverslips and allowed to dry for ∼12–18 h on a warming tray before being affixed onto microscope slides using Cargille Meltmount™ 1.704 (Cargille, Wayzata, MS, USA). The slides were examined under an Olympus BX51 microscope at 1,000x oil-immersion magnification (NA 1.25), to identify chrysophyte scales and diatom valves to species level, with statistically significant populations of ∼400 scales and ∼600 valves counted for each (Patterson & Fishbein, 1989).

The siliceous remains of certain species that did not present easily-observable, taxon-defining ornamentation under standard light microscope conditions were verified using scanning electron microscopy (SEM). For SEM analysis, subsamples from previously prepared sample slurries were pipetted onto aluminum foil or glass coverslips, dried and mounted onto scanning electron microscope stubs using double-sided carbon tape. The sample stubs were then coated with gold-palladium using a Denton DESKII sputter coater. The sample stubs were examined using either the Tesca VegaII XMU SEM at the Nano Imaging Facility at Carleton University or the FEI Apreo SEM at the Canadian Museum of Nature (Ottawa, Canada) using images created by both secondary and back-scatter electrons. SEM working distances ranged from 3–10 mm under 5KV. Scales and frustules were identified using images and descriptions found within the taxonomic literature and the SEM image collection at the Canadian Museum of Nature (Siver, 1991; Krammer & Lange-Bertalot, 1988; Houk, Klee & Tanaka, 2010; Siver & Hamilton, 2011; Alexson et al., 2022; Guiry & Guiry, 2022). The chronology of examined samples was varve-year inferred by counting from known events such as the thick layers associated with the Dust Bowl. Samples and slides are deposited at the national phycology collection at the Canadian Museum of Nature (CANA 129343-129391).

### Statistical analysis

Species abundance of the statistically significant taxa were plotted using Tilia *v.* 2.0.2 (Grimm, 1987). A stratigraphically constrained cluster analysis (CONISS) was performed on the combined chrysophyte scale and diatom valve relative abundance data. This analysis provides clusters based on an incremental sum of squares method and Euclidean distance, considering only stratigraphically adjacent samples to better represent groups through the history of the sediment record (Grimm, 1987). Separate zones marked through CONISS were validated with a broken-stick model using the *rioja* package (Juggins, 2020) within R studio (R Core Team, 2020). Non-metric multidimensional scaling (NMDS) was performed on non-transformed and square-root transformed species abundance data, using Bray–Curtis Dissimilarity as distance within R studio using the vegan package (Oksanen et al., 2020), to view assemblage and species data with reduced dimensions, and allow for visualization of data within a two-dimensional graphical interpretation; as such, the NMDS bi-plot was a second metric along with CONISS to identify and visualize groups in a reduced dimensional space.

## Results

**Figure 3:**
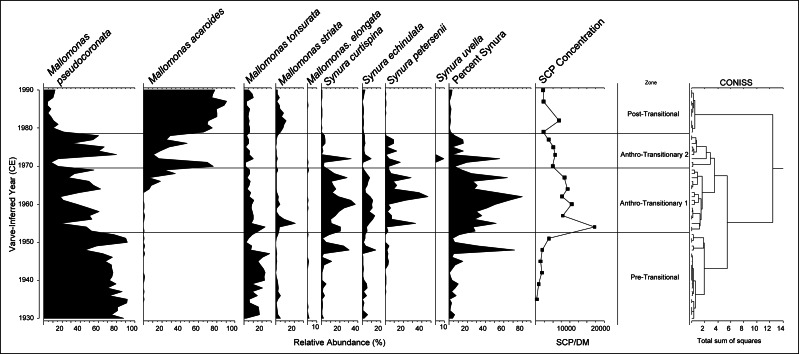
Relative abundance of common chrysophyte taxa within Crawford Lake by varve-inferred year (CE). Percentage of scales belonging to *Synura* by year depicted. SCP concentrations in number per gram of sediment dry mass (gDM^−1^) and plotted according to median sample year; from McCarthy et al. (2023). Zones created by broken-stick model validated CONISS delineations based on counted chrysophyte scale data.

Twelve scaled chrysophyte taxa were identified within the core taken for this study, of which seven reached abundances of >10% in at least two samples. In total, 371 diatom taxa were identified during counting with most being rare temporally. Sixty taxa and eight general genera taxa groups had >10 valves present in at least one sample and were selected based on this criterion. A broken-stick model validated CONISS analyses which identified four distinct paleoenvironmental units within the scaled chrysophytes and three distinct zones within the diatom communities (Figs. 2 and 3). Within the chrysophytes, these zones were identified as the: Pre-Transitional Zone (1930–1952); Anthro-Transitionary Zone 1 (1953–1969); Anthro-Transitionary Zone 2 (1970–1978); and Post-Transitional Zone (1979–1990). Diatom assemblages showed three zones spanned across 1930–1941, 1942–1968, and 1969–1978 and were identified as the Pre-Transitional Zone, Anthro-Transitionary Zone, and Post-Transitional Zone, respectively.

The Pre-Transitional zone had only one year (1949) with an average monthly summer air temperature >18.5 °C and four years with temperatures >18.0 °C (Supplement 3, regional weather data). Heavy summer rainfall levels >450 mm were observed three years through the period 1938–1945 and total snowfall from November–March exceeded 175 cm in 1939, 1942, 1944, and 1950. The Anthro-Transitionary Zone 1 experienced two years (1955, 1959) with average monthly air temperatures exceeding 19 °C and were characterized by 2 years (1952, 1968) with summer rains exceeding 450 mm and no years where snow exceeded 200 cm. Anthro-Transitionary Zone 2 had no years with average summer air temperatures >18 °C, two years (1973, 1976) with summer rains >450 mm and one year (1972) with high snow accumulation. Finally, the Post-Transitional Zone had two years of higher monthly temperatures (1987, 1988), two years of elevated summer rainfall (1979, 1985) and no years with snowfall >200 cm.

### Chrysophyte-delineated zones

The 1930–1952 Pre-Anthropocene Zone was characterized by a large proportion of *Mallomonas pseudocoronata* Prescott; *Mallomonas tonsurata* Teiling being the second most dominant species of the interval. Combined, these two species generally provide more than 90% of the scales counted in this zone.

Anthro-Transitionary Zone 1 was defined by a large and continuous increase of *Synura* species, *Synura curtispina* Asmund, *Synura echinulata* Korshikov and *Synura petersenii* Korshikov, changing from <10% abundance in most years within the Pre-Anthropocene Zone to becoming the species present in highest abundance. This zone also captured the large increase in SCP concentration within the sediments of Crawford Lake (Figs. 2 and 3).

Anthro-Transitionary Zone 2 (1970–1978) was defined by the spike of *Mallomonas acaroides* Zacharias *sensu* stricto, followed by a community shift to *M. pseudocoronata* with a decrease of *Synura* species compared to the previous zone. Between the shift from *M*. *acaroides* to *M*. *pseudocoronata*, there was a short notable increase in *S*. *curtispina* and *S*. *petersenii* (Fig. 3). Within this interval, SCP concentration began to decline from the high present within the previous zone and had the lowest relative abundance of *Mallomonas striata* Harris and Bradley sensu stricto, which was present in all zones.

The Post-Transitional Zone, characterizing Crawford Lake from 1979 to 1990 had a total dominance of *M. acaroides* (>70% relative abundance) with minor populations of *M. striata*, *M. pseudocoronata* and *M. tonsurata*. *Synura* species made up less than 5% scale abundance through this time interval.

**Figure 4:**
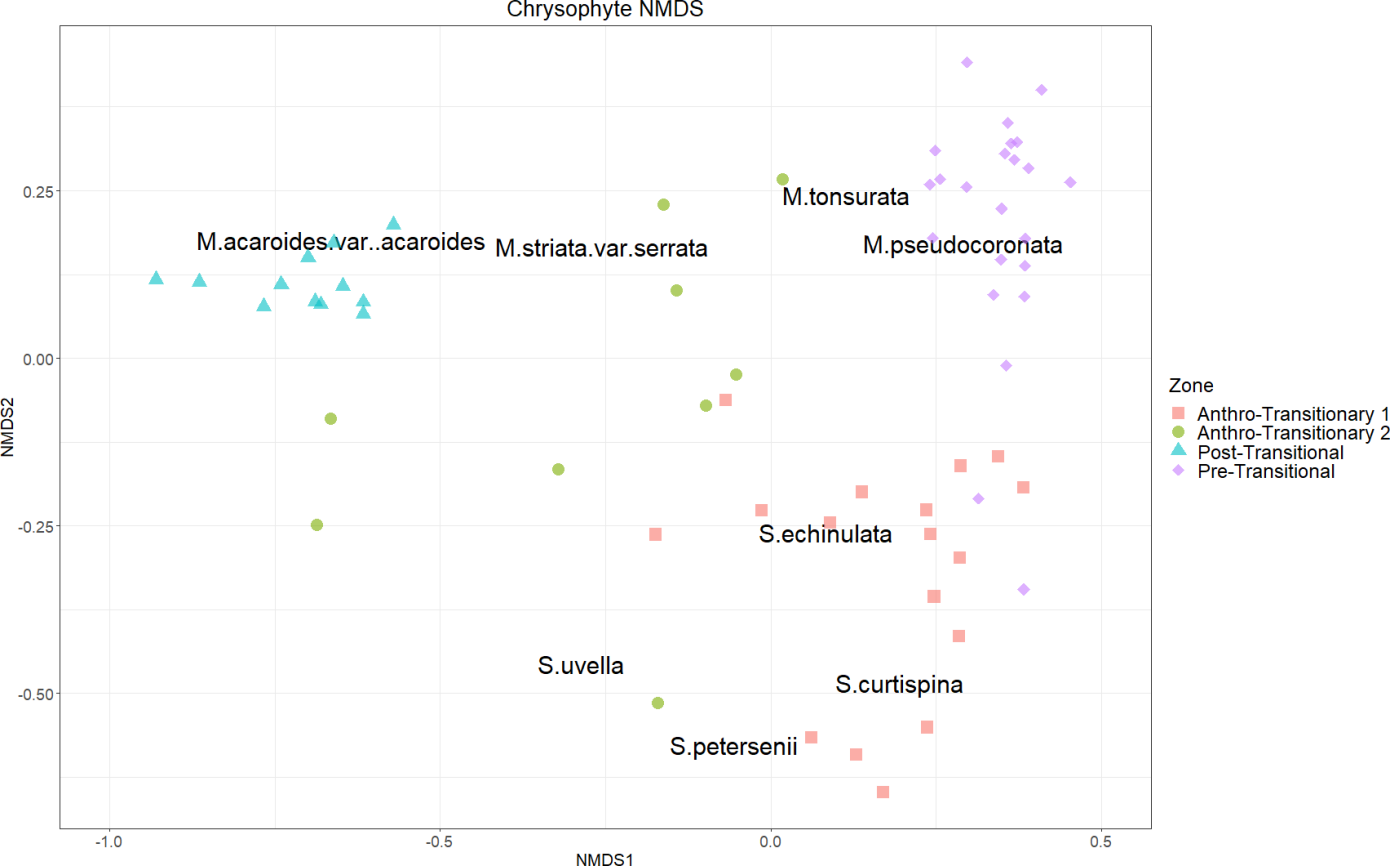
Chrysophyte NMDS. NMDS biplot based on square-root transformed chrysophyte scale count data (*k* = 3, stress = 0.061009). CONISS delineated zones marked with colours and symbols as depicted in the legend.

Non-metric multidimensional scaling (NMDS) analysis showed the Pre-Transitional Zone plotting in the same space as *M. pseudocoronata* and *M. tonsurata*, Anthro-Transitionary Zone1 plotted with *S*. *echinulata*, *S. curtispina* and *S. petersenii*. The zone Anthro-Transitionary 2 formed a broad scatter having no tangible species alignments with the exception of a distant association of *S. uvella* with one assemblage (Fig. 4). The Post-Transitional Zone assemblages plotted together showing a clear association with *M. acaroides*.

### Diatom-delineated zones

**Figure 5:**
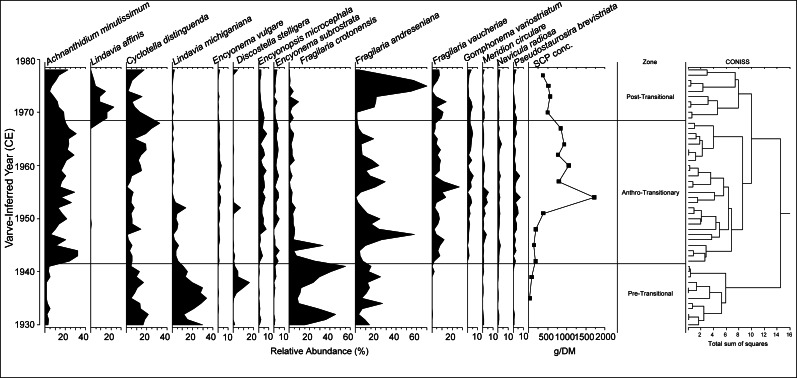
Relative abundance of common diatom taxa within Crawford Lake by varve-inferred year (CE). SCP concentrations in number per gram of sediment dry mass (gDM^−1^) and plotted according to median sample year; from McCarthy et al. (2023). Zones created by broken-stick model validated CONISS delineations based on counted diatom valve data.

From 1930–1941, the Pre-Transitional zone comprised *Fragilaria crotonensis* Kitton, *Lindavia michiganiana* (Skvortzov) T.Nakov et al., and *Discostella stelligera* (Cleve & Grunow) Houk & Klee at their highest relative abundances within the observed record, and generally made up the majority of observed valves (Fig. 5).

The Anthro-Transitionary Zone (1942–1968) featured a decrease in the relative abundance of *F. crotonensis*, a large increase in the relative abundance of *Cyclotella distinguenda* Hustedt and *Achnanthidium minutissimum* (Kützing) Czarnecki, as well as the appearance of *Fragilaria vaucheriae* (Kützing) J.B.Petersen in quantities above 5% relative abundance (Fig. 5). This Zone also showed increases of other tychoplanktic and benthic taxa such as *Meridion circulare* (Greville) C.Agardh, *Navicula radiosa* Kützing, and *Gomphonema variostriatum* K.E. Camburn & D.F. Charles. A subdivision of this zone was noted at 1962 with a subsequent general decline of *F. andreseniana* Alexson et al., and increases in *C. distinguenda* and *A. minutissimum*.

The communities within the Post-Transitional Zone (1969–1978) were mostly of the planktic and tychoplanktic species *Fragilaria andreseniana, Lindavia affinis* (Grunow) Nakov, Guillory, M.L.Julius, E.C.Theriot & A.J.Alverson and *C. distinguenda*. Through this period there was a shift from *A. minutissimum*, *L. affinis* and *C. distinguenda* to *F. andreseniana* and then back to *A. minutissimum* and *C. distinguenda* (Fig. 5).

**Figure 6:**
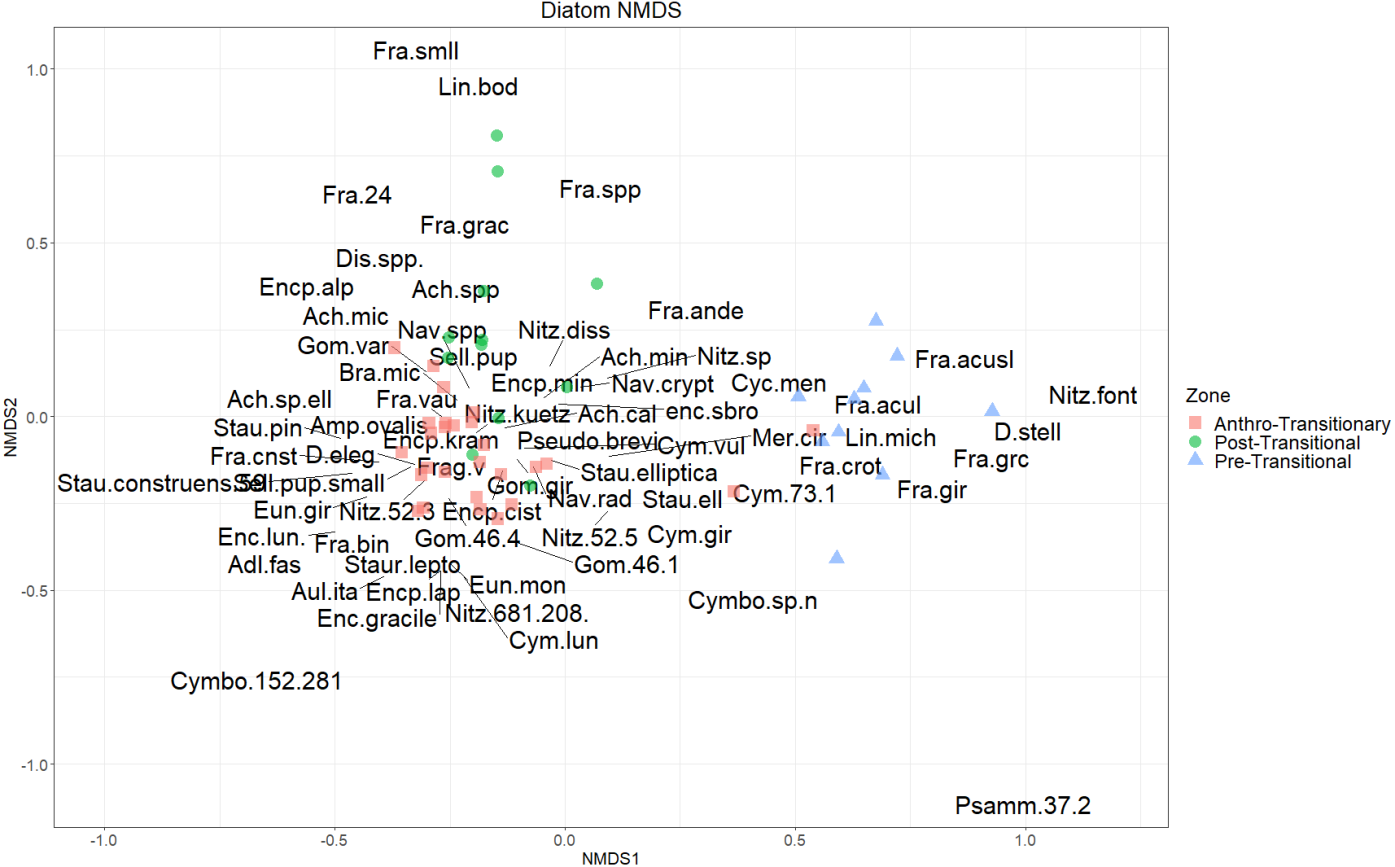
Diatom NMDS. NMDS biplot based on square-root transformed diatom valve count data (*k* = 3, stress = 0.09402618). CONISS delineated zones marked with colours and symbols as depicted in the legend. Taxonomic names of diatoms matched to abbreviations within this figure are available in table form within Supplement 6.

NMDS of the diatom communities showed the Pre-Transitional samples were within the same space as *F. crotonensis*, *Fragilaria* spp, *D. stelligera*, and *L. michiganiana* (Fig. 6). Assemblages from the Anthro-Transitionary Zone plotted toward Post-Transitional Zone but were still distinct and associated with many diatom species. Pre-1960, the assemblages contained small araphids (*Staurosira* spp, *Staurosirella* spp and *Pseudostaurosira brevistriata* (Grunow) D.M.Williams & Round), along with other benthic forms including *Encyonema* spp, *Adlafia* spp. and *Nitzschia* spp. Post 1960, the Anthro-Transitionary Zone plotted in the same space as the monoraphids (*Achnanthidium* spp), as well as the biraphid taxa; *Gomphonema variostriatum* and *Brachysira microcephalum*. Assemblages belonging to the Post-Transitional Zone were scattered along the second the second NMDS axis,, but are associated with species such as *L. affinis*, *F. andreseniana*, other *Fragilaria* spp, and *Nitzschia* taxa (Fig. 6)

## Discussion

Statistical analysis of the scaled chrysophyte population data in varved sediments from Crawford Lake shows three distinct paleoenvironmental shifts through the 1930–1990 interval resulting in four distinct assemblages, with the diatoms showing two distinct shifts through this same interval (1930–1978) aligning with three clear assemblages. Chrysophytes and diatoms highlight local, regional and possibly global (anthropogenic/climate) impacts on Crawford Lake. Using high-resolution annual subsampling afforded by the unique annually deposited varves that characterize sedimentation in Crawford Lake, these significant paleoenvironmental shifts are narrowed to precise year boundaries for each algal group. Both chrysophytes and diatoms identify community shifts during and immediately after the Second World War with the large-scale increases in industrialization across northeastern North America and in the context of Crawford Lake, within the Toronto-Hamilton region of Ontario (Arnold, 2007; Arnold, 2012).

Trends in SCP deposition, a proxy for the burning of fossil fuels and industrial emissions, found within the lake also show exponential increases during the 1940s before spiking around 1955 and then gradually declining to levels similar to those of the 1930’s and 40’s (Ruppel et al., 2013). Regional climate during this same period (1937–1952) had consistently average summer air temperatures and four years with high rainfall (>450 mm) possibly linked to SCP deposition.

General trends in the chrysophyte communities within Crawford Lake shift from *M. pseudocoronata*, the most abundant *Mallomonas* species over the early to mid-1900’s, to *M. acaroides* as the most abundant species in the late 1900’s, with *Synura* species, dominating the Anthro-Transitionary period from 1952–1978. *Synura* is known to have colony-forming species that bloom on or under the thermocline within lacustrine systems with high abundances observed through the mid-twentieth century (Siver, 2003; Mushet et al., 2017). Within the diatoms, the communities characterizing the early 20th century record in Crawford Lake are composed mainly of planktic species such as *C. distinguenda*, *F. crotonensis*, *L. michiganiana*, and *F. andreseniana*. These communities change during the Anthro-Transitionary period (1951–1952 low to high shift in summer rainfall, Supplement 3) to assemblages more heavily favoring disturbance tolerant tychoplanktic and benthic species such as *F. vaucheriae*, *A. minutissimum*, *M. circulare*, and *G. variostriatum*. Through the 1968–1969, shift to Post-Transitional Zone, there was a high to low change in summer rainfall (Supplement 3) with an assemblage shift back to planktic species, although not similar to pre-1943, fluctuating between centric diatoms and the araphid *F. andreseniana*.

### Initial anthropogenic transition (1942-1968)

Through the 1930s prior to the transition, the scaled chrysophytes showed little to no alteration in the community assemblage, while the planktic dominant diatom community fluctuates between centric diatoms and the pennate *Fragilaria crotonensis*, associated with changes in productivity (Hutchinson, 1967). Although *Synura* species are found lower within the water column comparatively to those of the *Mallomonas* genus, all these species require sunlight to maintain photosynthetic activities needed for survival. As such, the observed increases, and eventual decreases in *Synura* taxa, is concomitant with the massive changes in SCP deposition, and reduced calcite precipitation, which are most likely associated with changes in light-attenuation regimes within the waters of Crawford Lake brought about through a regional weather and a reduction of humic substances within the water in response to acidification related to industrial emissions (Monteith et al., 2007; Hruška et al., 2009; Llew-Williams, 2022). Through this period into the early 1950s elevated summer rainfall (5 years) possibly enhanced local terrestrial loading with four years of rainfall >450 mm. Dilution of surface waters at this time is evidenced by very thin light-coloured laminae, since warm summer temperatures and high pH (high concentrations of Ca^+2^ and CO_3_^−2^ ions) are required for precipitation and accumulation of calcite crystals (Llew-Williams, 2022; McCarthy et al., 2023).

Diatom community changes occurred in the early 1940s with a shift from planktic species to tychoplanktic and benthic; this shift to deeper-dwelling species associated with these waters increasing in clarity (Ekdahl et al., 2004; Gushulak et al., 2021). From 1941 to 1942, there was a dramatic increase in summer storms (262 mm to 479 mm of accumulation) in the region creating local impacts to the lake. Regional storms likely affected terrestrial loading to the lake with the observed switch from planktic to tychoplanktic and benthic species. Although not linked directly to peak carbon particle concentrations, changes in the diatom community occur with the first stepwise increase of SCPs also suggesting the lake environment was changing. SCP terrestrial deposition from previous years could be impacting SCP deposition rates in the lake with the more frequent summer storms increasing loading through the 1940s, which may further impact algal communities with an increased availability of heavy metals related to the sources of SCP deposition (McCarthy et al., 2023). The diatom community was further characterized by two sub-assemblages during the transition period, pre- and post-1962 (Fig. 6). Diatom assemblages in a constrained CONISS analysis shows a species shift after 1962 (Fig. 5). Community assemblages are changing through time, but the significance of these changes is unclear, although average monthly summer air temperatures (May-September) were elevated (>18 C) from 1959 to 1961 with 1959 temperatures reaching 19.8 C, the highest levels during the Great Acceleration in the region. *Achnanthidium minutissimum* and *C. distinguenda* are the primary species in the pre- and post- 1962 shift. Likewise, *Synura* species are increasing and then declining during the same period. Markers of global anthropogenic signatures on Crawford Lake would include plutonium (Pu) from the peak of atomic testing during the transition period. McCarthy et al. (2023) show Pu^239,240^ concentrations in the lake sediments increase through the early 1950s, peak in 1963 and then decline. Although Pu and algal assemblages are not causally correlated, the metrics both show a similar trend that can be linked to a time in which the lake was documenting and also impacted by global anthropogenic stressors, and climate.

The probable driver for the shift in chrysophyte assemblages to favoring *Synura* over *Mallomonas* as well as the changes within diatoms to favor a higher relative abundance of benthic species is believed to be weather patterns with increased industrial emissions and acidic deposition on the lake catchment. These changes result in reduced transport of humic substances into the water column, and increasing light penetration within the waters. This is brought about by WWII and post-WWII industrialization, including increased ore smelting and steel production within the Toronto-Hamilton regions and further across eastern North America (Arnold, 2007; Arnold, 2012). Associated with more industry is increased human settlement, urban expansion, and higher motor vehicle usage, all of which would be associated with the observed increase in atmospheric SCPs and deposition within the area (Fig. 2; Steffen et al., 2011; Rose, 2015; McCarthy et al., 2023). The size fractionation of SCPs through the study show the prominence of 10–25 µm carbon particles throughout the ∼150 years of analysis (Fig. 2). However, during the Great Acceleration (1940–2010) smaller particles (<10 µm) appear and persist, comprising <20% of the contamination, reflecting longer distance transport. The large particles that are relatively common (albeit in low total concentrations) earlier are probably derived from local burning of coal. Accumulating SCP and smelting gases (*e.g.*, sulfur) create the potential for lake acidification. Crawford Lake is an alkaline buffered system that should withstand atmospheric acidification events. Despite this, like many other bodies of water with well-buffered catchment areas (Hruška et al., 2009; Meyer-Jacob et al., 2020), Crawford Lake shows changes implying acid impacts.

Although Crawford Lake is a highly buffered system overlaying a dolomitic limestone base, it is most probably regional weather and impacts of acid deposition on the catchment of the lake that are probably the most significant drivers behind the inferred changes to the water clarity and sediment/particulate loading. Acidification impacts on the lake may have been amplified through chemical interactions and the binding of organic humic substances which would contribute to the reduction of dissolved organic carbon (DOC) reaching the lacustrine system (Monteith et al., 2007; Hruška et al., 2009). The peak of acid deposition within this region occurred by 1970 and was due primarily to large emissions of sulfur and nitrogen oxides from industrial emitters both locally in Ontario and from upwind sources in the industrial heartland of the US; this trend is mirrored in the observed deposition of SCPs within Crawford Lake (National Air Pollution Surveillance Program, 2020; National Pollutant Release Inventory, 2022).

Biologically, studies have found similar trends in *Synura* species abundance changes related to DOC content within the water column (Hadley, 2012). Like scaled chrysophytes, the diatom shifts from planktic to littoral and benthic taxa, along with the recognition of disturbance tolerant species like *A. minutissimum* (Jüttner et al., 2022), indicate water clarity, along with less obvious factors played a role in the 1940’s anthropogenic shift. During the Anthro-Transitionary period when weather and anthropogenic acid deposition was impacting Crawford Lake, reforestation was also occurring in the immediate area, which may have contributed to increasing water-clarity through reduction in erosion and sediment export into connected waters (Keller & Fox, 2019).

### Post-transition (1968-1990)

The later shifts in assemblages to post-Transitional are hypothesized to have been caused by the opposite effect; the lake waters becoming less clear due to local stressors and a reduction in acid deposition within the region caused primarily by emissions legislation changes. During the 1968–1969 transition period, regional summer rain levels dropped from 408.9 to 293.3 mm indicating fewer storms and possibly higher lake productivity. Also, at this time the acquisition of the Crawford Lake area into lands protected by Conservation Halton was completed with educational related site construction activities (Meyer-Jacob et al., 2019; Meyer-Jacob et al., 2020; National Air Pollution Surveillance Program, 2020). The change from this Anthro-Transitionary period of clear water termed by some as “water re-browning”, has been reported from other well-buffered, as well as chronically acidic lakes, that suffered from similar anthropogenic impacts (*e.g.*, Meyer-Jacob et al., 2019; Meyer-Jacob et al., 2020).

The large assemblage shift that occurred in the 1970’s coincides with the re-emergence of *Mallomonas* species in high abundances within the chrysophytes and an increase in centric planktic species within the diatom community. Although this change was in part brought about by water re-browning after recovery from acid impacts, it may also have been impacted by Crawford Lake’s incorporation into the Crawford Lake Conservation Area with boardwalk construction around the lake initiated in the late 1960s along with land alteration near the park in 1969 as well as a fire that destroyed the adjacent Crawford family home around the same interval (Muller, 1982; B. Bartley, Halton Conservation, 2020, pers. comm.) In addition, the region experienced a clear decline in rainfall between 1968 and 1969 (Supplement 3, Environment Canada, 2022). The diatom assemblage shift, at this time, was the second most important during the Great Acceleration (Fig. 5). In light of the continued occurrence of *A. minutissimum* at the beginning of the Post-Transition, then decline over the 1970s, as well as the appearance of *L. affinis*, we suggest that regional weather and boardwalk construction (local anthropogenic impact) in the late 1960s assisted in the observed transition. This transition was associated with increasing water turbidity and amplifying the re-browning of lake waters, which in turn reduces the amount of light benthic taxa such as *A. minutissimum* have for photosynthetic activities. The shift to *M. acaroides* at the Anthro-Transitionary 2, followed by a sharp shift to *M. pseudocoronata*, then shift back to favouring *M. acaroides*, also supports the observation of weather and a local anthropogenic disturbance.

The Post-Transitional change in scaled chrysophytes back to a dominance of *M. acaroides* during the 1980s is not aligned with annual rainfall or seasonal air temperatures, but is associated with Conservation Halton’s second phase of construction activities in the early 1980s, in which a boardwalk was completely constructed around the lake (B. Bartley, Conservation Halton, 2020, pers. comm.). The planktic diatom *Asterionella formosa* Hassall appeared for the first time during the Post-Transition showing changes in lake eutrophication status associated with disturbance (*e.g.*, Reynolds, 1984). The dominance of *M. acaroides* throughout the 1980s clearly shows a consistent longer-term change in the lake community. It is important to note, that changes in abundance of *Synura* species, and replacement of *M. pseudocoronata* with *M. acaroides* have been observed within lakes elsewhere in Canada, some of which are considered minimally impacted, and may be resultant from changes in CO_2_ regimes brought about by climate change (Mushet et al., 2017; Mushet et al., 2018; Wolfe & Siver, 2013).

The documentation of weather and anthropogenic stressors at the local, regional and likely global scale show the sensitivity of, and impact on Crawford Lake. Climate change and water chemistry shifts brought about by anthropogenic impacts, as well as similar trends being seen in algal assemblages elsewhere, indicate the preserved communities within the sediments of Crawford Lake are representative of regional (eastern North America) weather and industrialization trends as well as the associated anthropogenic impacts on the global environment. The historical sediment record documenting and assessing past anthropogenic impacts by First Nations cultures further supports the sensitivity of the Crawford Lake ecosystem to external stress across long term spatial and temporal scales.

## Conclusions

The varved sediments of Crawford Lake have been used to identify changes within the contained community of siliceous algae at a highly precise resolution. The sediments record large changes within the communities of siliceous algae, particularly during the mid-20th century; scaled chrysophytes showing a response to water clearing and re-browning in response to likely regional weather and acid impacts from 1952 to 1969, with massive shifts between *Mallomonas* (pre-1950, post-1970) and *Synura* (1950s–1960s) taxa. The diatom assemblages, predominantly planktic, show finer temporal fluctuations starting in the early 1940s with a shift between *Fragilaria crotonensis* and *F. andreseniana*, while the littoral monoraphid *Achnanthidium minutissimum* persistent from the 1940s to 1970. Overall, the changes of these algal communities, recorded in the sediments of Crawford Lake, are concurrent with elevated annual rain events or air temperature, coupled with the changes in deposition of SCP and plutonium in the mid-20th century. Furthermore, despite regulations resulting in the decreased deposition of these anthropogenic markers, the more modern communities of siliceous algae have changed in composition from those of the early 20th century, likely because of regional climate and local anthropogenic activity.

This small, contained lake system presents clear evidence for the Great Acceleration showing the broad and deep influence a global shift (including shifts at a subcontinental scale) can have on lake systems throughout the world. This has significant implications for policy makers and planners as they develop policies for water resource management and ecosystem protection. The sensitivity of Crawford Lake to anthropogenic impacts at local, regional, and global scales and the extensive historical data captured in the sediments (marking anchored anthropogenic activities) makes this lake a good candidate to support a mid-20th century lower boundary for the proposed Anthropocene epoch.

##  Supplemental Information

10.7717/peerj.14847/supp-1 Supplemental Information 1 Raw chrysophyte siliceous microfossil counts

10.7717/peerj.14847/supp-2 Supplemental Information 2 Raw diatom siliceous microfossil counts

10.7717/peerj.14847/supp-3 Supplemental Information 3 Toronto Airport Climate Data Summary of weather data for the TORONTO LESTER B. PEARSON INT’L A weather station (6158733, Long.: -79.63, Lat.: 43.68) from 1938–1990. Data retrieved from Environment Canada, 01-Nov-2022 ( https://climate.weather.gc.ca/)

10.7717/peerj.14847/supp-4 Supplemental Information 4 Diatom and Chrysophyte R Code R code for NMDS graphic creation as well as broken-stick model CONISS Zone validation.

10.7717/peerj.14847/supp-5 Supplemental Information 5 Spreadsheet displaying SCP concentrations and sizes within 2019 Crawford Lake sediment samples Left: SCP counts and concentrations by sample number and varve-inferred date. Right: SCP size fraction percentage with graphical representation.

10.7717/peerj.14847/supp-6 Supplemental Information 6 Diatom species displayed within NMDS Abbreviations displayed within Fig. 6 with accompanying genus and species name. Notes are in the final column.

## Additional Information and Declarations

### Data Availability

The following information was supplied regarding data availability: Annual resolution siliceous microfossil counts are available in the Supplementary Files.
